# In Patients with Obesity, Are Affective Temperaments Associated with Attrition? An Evaluation during and before the SARS-CoV-2 Pandemic

**DOI:** 10.3390/jcm11030862

**Published:** 2022-02-07

**Authors:** Enrica Marzola, Giovanni Abbate-Daga, Elena Scumaci, Valentina Ponzo, Ilaria Goitre, Marianna Pellegrini, Chiara D’Eusebio, Andrea Benso, Sara Belcastro, Franco De Michieli, Chiara Crespi, Fabio Broglio, Ezio Ghigo, Simona Bo

**Affiliations:** 1 Eating Disorders Center, Department of Neuroscience “Rita Levi Montalcini”, “Città della Salute e della Scienza” Hospital of Turin, University of Turin, 10126 Turin, Italy; enrica.marzola@unito.it; 2 Diabetes and Metabolic Diseases Unit, Department of Medical Sciences, “Città della Salute e della Scienza” Hospital of Torino, University of Torino, 10126 Torino, Italy; elena.scumaci@ibero.it (E.S.); valentina.ponzo@unito.it (V.P.); ilaria.goitre@libero.it (I.G.); marianna.pellegrini@unito.it (M.P.); chiaradeusi@gmail.com (C.D.); andrea.benso@unito.it (A.B.); sara.belcastro@unito.it (S.B.); franco.demichieli@unito.it (F.D.M.); chiaracrespi@hotmail.it (C.C.); fabio.broglio@unito.it (F.B.); ezio.ghigo@unito.it (E.G.)

**Keywords:** attrition, cyclothymic, drop-out, obesity, temperament

## Abstract

Timely data on attrition from weight loss programs for patients with obesity during the SARS-CoV-2 pandemic are lacking, so we aimed to contribute to filling this gap in the literature by comparing attrition during or outside of the SARS-CoV-2 pandemic and its possible association with patients’ affective temperaments, psychopathology, and clinical variables. Two-hundred and eleven outpatients with obesity were recruited and completed the Temperament Evaluation of Memphis, Pisa, and San Diego Auto-questionnaire, Binge Eating Scale, Beck Depression Inventory, and State-Trait Anxiety Inventory. Those who dropped out during the pandemic period were mostly men, with younger age of weight gain, and with a larger waist circumference than completers. Patients with obesity who dropped out outside of the SARS-CoV-2 pandemic showed marked levels of depression, anxiety, binge eating episodes, and higher affective temperaments (but the hyperthymic one) when compared to their counterparts. The cyclothymic temperament slightly increased attrition (OR = 1.13, 95% CI 1.00–1.27 *p* = 0.05) outside the pandemic, while during the pandemic, male gender (OR = 3.50, 1.04–11.7, *p* = 0.04) was associated with attrition. These findings suggested that male patients with obesity are at particular risk of drop-out from weight-loss treatment during the SARS-CoV-2 pandemic; contrariwise, outside the pandemic, affective temperaments could be a useful baseline assessment for defining the attrition risk in these patients.

## 1. Introduction

The SARS-CoV-2 pandemic has recently proposed a reconceptualization of obesity (and obesity-related metabolic disorders) as a pandemic in the pandemic since it has been authoritatively stated how patients with obesity and metabolic disorders had to face a much hard situation [1] because of a wide array of reasons: First, access to care has been reduced because of the well-known decreased access to primary care providers and the need to reallocate hospital resources [1]. Secondly, obesity is associated with the risk of SARS-CoV-2-related death [2] and represents an independent risk for hypoventilation syndrome—notwithstanding challenging intubation—in Intensive Care Unit (ICU) patients [3], thus contributing to respiratory failure in patients with acute respiratory distress syndrome [4]. Thirdly, the SARS-CoV-2 outbreak itself entailed a severe mental health burden, mostly in those countries that were strongly hit [5], and this could be of importance in the field of obesity, frequently accompanied by sub-threshold affective conditions [6]. Finally, containment measures and fears of infection could have enhanced patients’ social isolation, potentially triggering obesity stigma [7].

Treating obesity is challenging, and attrition from weight loss programs is a hallmark of individuals with obesity, being as high as 80% even before the SARS-CoV-2 outbreak [8,9]. Patients who tend to drop out have been commonly described as being younger; reporting other medical comorbidities in addition to obesity; having a high BMI (frequently coupled with failed previous attempts to lose weight); being of low socioeconomic status, job status, or commitment; distance between home and the obesity treatment center; and lack of social support. A wide number of factors has been studied in the field of attrition in obesity, too, such as failure to lose weight, previous attempts at obesity treatment, significative expectation of success, levels of stress, alexithymia, and perceived loneliness, with depression also being associated with attrition for early dropouts [9]. Additionally, body dissatisfaction and the presence of binge eating episodes also represent attrition-related factors.

In this light, the association between personality traits and attrition from weight loss programs in patients with obesity has been previously studied, although with less consistent results because of the variety of personality assessments performed. Studies adopting a biopsychosocial perspective found low novelty-seeking and high self-directedness as predicting factors for successful short-term weight loss outcomes (≤6 months), while low novelty-seeking and high persistence seem to predict better weight loss maintenance, especially after bariatric surgery [10]. On the other hand, the influence between attrition and personality is much more controversial [10,11].

Over the course of the years, other models of personality have been proposed in addition to the well-known biopsychosocial model of personality. In particular, affective temperaments have been proposed as those biological and genetically-based traits of personality that represent our affective basic vulnerability to the environment. The Nineteenth-century psychiatrists Kraepelin and Kretschmer proposed that full-blown affective pathology and premorbid temperaments could shift on a continuum of intensity. Along these lines, Akiskal and collaborators proposed more recently a model by which five temperaments exist: depressive, hyperthymic, cyclothymic, irritable, and anxious. Such an investigation of personality has been increasingly used in psychiatry [12,13,14,15], as well as in other fields of medicine [16,17,18]. Recently, cyclothymic and depressive temperaments have been associated with an increased risk of moderate-to-severe psychological distress following the SARS-CoV-2 outbreak [19]. A previous study reported marked scores on the cyclothymic, irritable, and anxious temperaments in patients with obesity [20]. Additionally, an association between the cyclothymic temperament and the risk of binge eating and a protective effect of hyperthymic temperament on both binge eating and multiple weight cycling have been suggested [16].

Nevertheless, to the best of the authors’ knowledge, no studies so far investigated the potential association between affective temperaments of outpatients with obesity in a real-world setting and their risk of attrition by taking into account the limitations imposed by the lockdown due to the SARS-CoV-2 infection. With more detail, we designed this study to (a) investigate if patients with obesity who dropped out during the SARS-CoV-2 pandemic (i.e., seeking treatment from March 2020 until March 2021) differed from those who dropped out outside the pandemic (i.e., seeking treatment before March 2020) with respect to anxiety and depressive symptoms and binge eating episodes; (b) ascertain whether patients’ baseline characteristics and affective temperaments could be predictors of attrition from the weight loss program, in or outside the course of the SARS-CoV-2 pandemic.

## 2. Materials and Methods

This was a retrospective study.

All patients who sought treatment at our obesity unit (Endocrinology Division of the “Città della Salute e della Scienza” Hospital of Torino) from January 2018 until December 2020 were consecutively included. Inclusion criteria were the following: (a) age 18–75 years; (b) BMI ≥ 30 kg/m^2^ and <45 kg/m^2^; and (c) being followed-up for at least 4 months at our obesity unit. Exclusion criteria were: (a) secondary causes of obesity; (b) previous bariatric surgery; (c) known psychiatric disorders diagnosed by an M.D. (including known eating disorders); (d) substance abuse within the last 12 months; and (e) pregnancy.

Patients with a psychiatric illness or secondary/more severe obesity were excluded because attrition in these patients could be linked to the underlying disease and/or concomitant therapy or the weight-loss programs may be different, respectively, with the consequent presence of potential confounding factors. Sixteen patients refused to be enrolled or returned incomplete assessments.

All participants gave their informed consent for the collection and analysis of their data.

The study was approved by the local Ethics Committee and was conducted following the principles of the Declaration of Helsinki.

### 2.1. The Weight Loss Program

Patients were offered 6 meetings over 12 months, with further visits eventually planned according to individual needs. During the scheduled visits, patients received both group sessions focusing on nutritional education and improvement of both awareness and self-control, and individual visits by endocrinologists, psychologists, and dieticians. All patients were offered an individually prescribed diet in accordance with the Mediterranean diet composition; an energy restriction could apply on the basis of individual caloric requirements and usual assumptions. Written and verbal recommendations were given to all patients, by focusing on practical lifestyle tips.

During the SARS-CoV-2 pandemic, starting from March 2020, group sessions were abolished, and all visits were carried out on an individual level. In particular, during the period of strict lockdown (i.e., during March, April, October, and November 2020), most visits were carried out remotely, through telemedicine.

The weight loss target was individualized and discussed with the patient. Generally, a 10% weight loss at 6 months was proposed. However, more or less stringent goals were suggested based on individual needs. All patients were followed for at least 1 year, even if they had achieved their weight loss goal before the end of the program.

### 2.2. Assessments

Our weight loss program has been previously described [9,16]. Weight and height were measured with the participants wearing light clothes and no shoes, respectively, by a mechanical column scale (SECA model 711, Seca GmbH & Co, Hamburg, Germany) and a Stadiometer (SECA 220 measuring rod, Seca GmbH & Co, Hamburg, Germany). Waist and neck circumferences were assessed by a plastic tape meter at the umbilicus level or under the cricoid cartilage, respectively.

All patients completed the following questionnaires:

- Temperament Evaluation of Memphis, Pisa, and San Diego Auto-questionnaire, Italian version (TEMPS-A [21]): It is a self-report instrument of 110 (109 for males) items assessing the following affective temperaments: hyperthymic, depressed, irritable, cyclothymic, and anxious;
- Binge Eating Scale, Italian version (BES [22]): A binge eating disorder was considered unlikely with scores of 0–16, possible with scores of 17–27, and likely with scores > 27;
- Beck Depression Inventory (BDI [23]): It is a questionnaire aimed at investigating depressive symptoms: Scores from 0 to 4 indicate minimal depressive symptoms, scores of 5–7 indicate mild depression, scores of 8–15 indicate moderate depression, and scores of 16–39 indicate severe depression;
- State-Trait Anxiety Inventory (STAI [24]) for both trait (stable) and state (current) anxiety. The anxiety scores range from 20 to 80, with higher values corresponding to higher anxiety.

The above psychometric questionnaires were checked and assessed by a trained psychologist.

### 2.3. Definitions

Patients who did not attend a scheduled appointment and declared they no longer want to participate in the program were considered to be dropouts [9]. In the present study, all the enrolled patients (from January 2018 until December 2020) were divided according to the period of attrition. The SARS-CoV-2 pandemic period started in Italy in March 2020. The dropouts of our obesity unit starting from March 2020 until March 2021 were considered as attritions during the pandemic period (SARS-CoV-2-drop group); the dropouts before March 2020 were considered as attritions outside the pandemic period (non-SARS-CoV-2-drop group).

Weight cycling was defined as intentional weight loss of ≥3 kg followed by involuntary weight regain of ≥3 kg in line with the literature [25,26]; patients experiencing ≥2 cycles were considered as multiple weight cyclers [26].

### 2.4. Statistical Analyses

Kolmogorov–Smirnov test was used to assess the distribution of the variables; the t-Student’s and chi-square tests, when appropriate, were used to evaluate between-group differences. Because the distributions of the BDI and the TEMPS-A scores were skewed, the Mann–Whitney test was employed to evaluate the between-group differences. A multiple logistic regression model was used to evaluate the associations between the risk of attrition, either overall or by the period of attendance to the obesity unit, and variables that resulted significantly different between groups (Statistica 10.0, StatSoft GmbH, Tulsa, Oklahoma, USA).

## 3. Results

### 3.1. Sociodemographic and Clinical Characteristics of the Sample

We analyzed 211 patients who met the inclusion criteria. Most were women and reported adverse events during their lifetime (e.g., bereavement, abortion, divorce, abandonment, job loss) (Table 1). More than 40% of the cohort was living alone, not working/retired, and reported multiple weight cycling. A high prevalence of episodes of binge eating was detected, even if the presence of a full-blown psychiatric disorder represented an exclusion criterion for this study.

### 3.2. Characteristics of Patients Who Dropped Out during and outside the Pandemic Period

Around 40% of participants received or should have received a visit to our obesity unit during the pandemic period (from March 2020 to March 2021). The characteristics of the patients visited during or outside the pandemic period are described in Supplementary Table S1. The former showed increased mental well-being, lower adverse events, and a higher degree of weight cycling (Supplementary Materials, Table S1).

The characteristics of patients who dropped out during and outside the pandemic period are described in Table 2. Overall, 45% percent of our patients dropped out: 39.3% during the pandemic period and 49.2% outside the pandemic period.

Those who dropped out during the pandemic period were more frequently males, had a larger waist, and lower age of first weight gain than those who did not drop out. Moreover, no differences in the psychological assessments emerged between those who dropped out during the pandemic and their counterparts (Table 2).

Outside the SARS-CoV-2 pandemic, patients who abandoned the course of treatment did not differ from those who did not dropout with respect to clinical variables; in contrast, those who dropped out outside the SARS-CoV-2 pandemic showed poorer scores on the psychological assessments than completers, reporting more depressive symptoms, trait-anxiety, and episodes of binge-eating, as well as higher scores on depressive, cyclothymic, irritable, and anxious affective temperaments (Table 2).

### 3.3. Predictors of Attrition

As shown in Table 3, concerning the predictors for the overall risk for attrition, in a multiple logistic regression analysis, only the TEMPS-A cyclothymic score was significantly associated with overall attrition (Table 3). During the pandemic period, male gender remained associated with the risk of attrition (Table 3). Outside the pandemic period, cyclothymic temperament showed a trend towards significance as a predictor of attrition (Table 3).

By comparing the two groups of patients who dropped out, scores for anxiety, depression, the corresponding TEMPS-A aspects, and binge eating were higher among those who dropped out outside the pandemic period (Supplementary Materials, Table S2).

## 4. Discussion

There is no doubt that the SARS-CoV-2 outbreak placed a heavy burden on individuals with obesity, and current data on attrition from weight loss programs during the SARS-CoV-2 pandemic are greatly lacking. Several main findings emerged from our study: First, individuals with obesity who dropped out during the pandemic period were more frequently men, with younger age of weight gain, and with a larger waist circumference than those who did not abandon treatments. Subjects who sought treatment during the pandemic reported fewer psychiatric symptoms than patients who sought treatment before the pandemic: Psychic factors probably influenced the choice and the possibility of access to treatment, too. Individuals with obesity who dropped out outside the SARS-CoV-2 pandemic period showed marked levels of depression, anxiety, binge eating episodes, and poorer scores on all the affective temperaments (but the hyperthymic one) when compared to their counterparts. Finally, the cyclothymic temperament significantly predicted attrition from the weight loss program, but when focusing on the pandemic period, only the male gender resulted to be a predictor of attrition.

The paucity of the data currently available makes it difficult to compare these findings to other studies. In line with previous studies on attrition in obesity [9], overall, the individuals who dropped out were more frequently males, showed a younger age of weight gain, and a larger waist circumference than completers. Interestingly, notwithstanding the SARS-CoV-2 outbreak, a substantial proportion of the patients was motivated enough to seek treatments with positive compliance during such a critical time.

No psychological differences emerged when comparing completers and non-completers during the pandemic, raising the question of whether the SARS-CoV-2 pandemic itself generated bias in the pool of individuals seeking treatment. This could suggest that the subjects with more mental problems had some difficulties in undertaking a treatment program during the pandemic. Indeed, the SARS-CoV-2 pandemic had notoriously accentuated stress, anxiety, and depression in the general population [27], particularly in subjects with previous psychiatric symptomatology [28]. The comparison between patients visited during and outside the pandemic period (Supplementary Table S1) confirmed that depression and anxiety may have impacted the search for treatment during the pandemic in subjects with more psychiatric symptomatology. In Italy, the overall affected cases reached 4,808,047 individuals and 132,391 people overall deceased (Ministry of Health update 8 November 2021 [29]); moreover, data reported by the Superior institute of health (update 5 October 2021) on 7910 deaths showed that obesity was present in 11% of cases [30]. Therefore, those who were motivated enough to seek help during such a critical period could be particularly motivated and “healthy” from a mental health perspective, not being representative of the general population. To the best of the authors’ knowledge, no data are currently available on attrition from weight loss programs during the SARS-CoV-2 pandemic, so future lines of research are warranted on these matters. It is noteworthy that male sex has been consistently described as a factor predicting progression to severe SARS-CoV-2 [31], so it could be surmised that male patients during the SARS-CoV-2 outbreak could have been more afraid to become infected, potentially interrupting treatments. Along these lines, it should be borne in mind that our treatment center is located in a hospital facility, thus potentially amplifying patients’ fears of the risk of contagion.

Relatedly, male gender was also the only predictor of attrition when focusing on the SARS-CoV-2 pandemic period. Our findings are consistent with earlier research reporting male gender as predicting attrition [32]; however, earlier literature associated males with a poor educational level, in turn, a proxy for patients’ socioeconomic status. Our data do not support any sociodemographic difference between completers and non-completers, so this finding needs further investigation. Again, as aforementioned, the male-related increased risk for SARS-CoV-2 infection [31] may have played a role in this regard.

In contrast, when taking into account people who dropped out outside the SARS-CoV-2 pandemic, no differences in baseline—either sociodemographic or clinical—variables emerged. However, those who dropped out reported significantly more compromised psychological profiles, with respect to anxiety and depressive symptoms, as well as binge eating episodes. In addition, all affective temperaments but the hyperthymic one were more markedly expressed. In this regard, our data are in line with earlier research reporting patients who drop out as more depressed, anxious, and prone to binge eating [9,16] than completers. Still, Amann and collaborators [20] also reported similar findings, with individuals with obesity reporting higher scores on all affective temperaments but the hyperthymic one when compared to healthy controls.

Concerning predictors of attrition outside the SARS-CoV-2 pandemic, it is noteworthy that the cyclothymic temperament resulted overall in being associated to a greater risk of drop out. This is of clinical interest since patients with obesity have been proposed to have a variety of subthreshold affective conditions [6], potentially making them more inclined to poor medication adherence [18]. It should also be borne in mind that the cyclothymic temperament could have a role in entailing sensation seeking and self-stimulating behaviors, thus contributing to poor patient compliance. In fact, mood instability could entail compliance instability. In fact, subjects with cyclothymic temperament are impulsive and might use food to control mood instability. Overall, the cyclothymic temperament is thought to underpin the relationship between obesity and mood disorders [6], so although our sample excluded patients with a full-blown psychiatric diagnosis, it cannot rule out that the cyclothymic temperament could have a role in making patients more prone to be less perseverant in pursuing their goals. Finally, it is crucial for clinicians to know that patients with cyclothymic temperament can develop subthreshold or full-phase bipolar disorders and use of antidepressants (such as fluoxetine) may be at risk of triggering a full-blown bipolar form in these patients [33].

In spite of several strengths, including the timely assessment of SARS-CoV-2-related clinical implications for obesity, the comprehensive assessment of multiple variables for all patients, and the “real world” setting, this study suffers from limitations, such as the small sample size deriving from a single-center, thus potentially jeopardizing the generalizability of our results. Furthermore, during the pandemic period, the approach to the patient changed, and during the strict lockdown, visits were carried out remotely, through telemedicine. This might have affected the results of the study. However, there is evidence that visits by telemedicine are as effective as in-person visits, and telemedicine is now considered a safe alternative that can successfully help patients with obesity in weight loss [34,35,36]. Lastly, the high scores of the Binge Eating Scale questionnaire we found in our patients suggest that some subjects could have minimized the eating symptomatology at the initial evaluation because of a sense of guilt, shame, or a lack of awareness of the eating problem, as described in literature [37,38].

Nevertheless, some potential clinical implications of these data should be considered. Since the SARS-CoV-2 pandemic is far from being over, male patients with obesity should be particularly supported in treatment, owing to their higher risk of attrition. Furthermore, an investigation of affective temperaments may be particularly welcomed as a preliminary assessment for patients with obesity who seek specialized treatments for weight loss.

In conclusion, our preliminary results outlined specific characteristics of individuals with obesity who dropped out from a weight loss program during and outside the SARS-CoV-2 pandemic and may be useful for the management of these patients in the clinical practice. Further studies on larger samples are warranted since data on this important issue are currently largely lacking.

## Figures and Tables

**Table 1:** Characteristics of the whole cohort and by the risk of attrition.

	Total Sample of People with Obesity *n* = 211			
	All	Attrition		*p*
	*n* = 211	Yes *n* = 95	No *n* = 116	
Clinical variables				
Age (years)	46.7 ± 15.3	44.5 ± 15.4	48.6 ± 15.1	0.06
Males (%)	21.8	24.2	19.8	0.44
Residing in a rural area (%)	17.5	21.1	14.7	0.22
Active smoker (%)	16.6	17.9	15.5	0.64
Living alone (%)	47.4	47.4	47.4	0.99
Not working/retired (%)	43.6	42.1	44.8	0.69
Secondary schools/graduation (%)	66.8	64.2	69.0	0.47
Adverse life events (%)	71.1	67.4	74.1	0.28
Multiple weight cycling (%)	49.3	50.5	48.3	0.74
Visits during the pandemic	42.2	36.8	46.6	0.16
Age of weight gain (years)	26.6 ± 13.9	24.6 ± 13.6	28.2 ± 13.9	0.06
Weight (kg)	98.8 ± 16.0	98.3 ± 16.5	99.2 ± 15.6	0.70
Height (cm)	163.8 ± 8.0	164.3 ± 8.1	163.4 ± 7.9	0.45
BMI (kg/m^2^)	36.8 ± 4.8	36.4 ± 5.1	37.1 ± 4.5	0.33
Waist circumference (cm)	114.6 ± 12.0	115.1 ± 11.7	114.1 ± 12.4	0.54
Neck circumference (cm)	38.0 ± 4.1	37.9 ± 4.2	38.1 ± 4.0	0.67
Psychological questionnaires				
BDI	9.4 ± 6.0	10.2 ± 6.2	8.8 ± 5.9	0.11 *
STAI-state	46.9 ± 11.9	46.5 ± 11.8	47.2 ± 11.9	0.67
STAI-trait	48.6 ± 11.0	49.2 ± 10.6	48.1 ± 11.4	0.48
BES	33.1 ± 10.4	35.3 ± 10.3	31.4 ± 10.1	**0.006**
TEMPS-A scores				
Depressive	10.7 ± 3.6	11.1 ± 3.7	10.4 ± 3.6	0.15 *
Cyclothymic	8.1 ± 4.6	9.4 ± 4.3	7.0 ± 4.6	<0.001 *
Hyperthymic	9.1 ± 4.6	8.9 ± 4.4	9.3 ± 4.9	0.58 *
Irritable	5.8 ± 4.2	6.4 ± 4.3	5.3 ± 4.0	0.044 *
Anxious	11.4 ± 5.6	11.8 ± 5.4	11.1 ± 5.8	0.38 *

* Mann–Whitney U-test; Legend: BDI: Beck Depression Inventory; STAI: State-Trait Anxiety Inventory; BES: Binge eating scale; TEMPS-A: Temperament Evaluation of Memphis, Pisa, Paris, and San Diego Auto-questionnaire.

**Table 2:** Attrition by the period of attendance to the obesity unit.

	Attrition during the Pandemic Period			Attrition outside the Pandemic Period		
	Yes *n* = 35	No Attrition *n* = 54	*p*	Yes *n* = 60	No Attrition *n* = 62	*p*
Clinical variables						
Age (years)	45.9 ± 14.1	49.6 ± 16.2	0.27	43.7 ± 16.1	47.6 ± 14.1	0.16
Males (%)	34.3	11.1	**0.008**	18.3	27.4	0.23
Residing in a rural area (%)	20.0	16.7	0.69	21.7	12.9	0.20
Active smoker (%)	14.3	20.4	0.47	20.0	11.3	0.18
Living alone (%)	37.1	51.9	0.17	53.3	43.6	0.28
Not working/retired (%)	40.0	50.0	0.36	43.3	40.3	0.74
Secondary schools/graduation (%)	65.7	74.1	0.40	63.3	64.5	0.89
Adverse life events (%)	51.4	70.4	0.07	76.7	77.4	0.92
Multiple weight cycling (%)	51.4	63.0	0.28	50.0	35.5	0.11
Age of weight gain (years)	23.4 ± 11.3	29.2 ± 14.4	**0.047**	25.2 ± 14.8	27.3 ± 13.5	0.41
Weight (kg)	98.8 ± 15.2	96.7 ± 13.4	0.49	98.1 ± 17.4	101.4 ± 17.1	0.29
Height (cm)	164.8 ± 8.2	162.8 ± 7.5	0.26	164.0 ± 8.1	163.9 ± 8.3	0.99
BMI (kg/m^2^)	36.4 ± 5.0	36.4 ± 4.4	0.96	36.4 ± 5.3	37.6 ± 4.6	0.19
Waist circumference (cm)	117.2 ± 12.5	111.9 ± 11.1	**0.038**	113.9 ± 11.1	116.1 ± 13.2	0.34
Neck circumference (cm)	38.1 ± 4.2	37.1 ± 3.8	0.26	37.8 ± 4.2	39.1 ± 4.0	0.09
Psychological questionnaires						
BDI	6.7 ± 5.7	8.6 ± 6.0	0.07	12.2 ± 5.6	9.1 ± 5.8	**0.002**
STAI-state	42.6 ± 10.9	46.7 ± 11.9	0.11	48.8 ± 11.8	47.6 ± 12.1	0.59
STAI- trait	43.6 ± 8.9	47.9 ± 10.9	0.06	52.4 ± 10.2	48.2 ± 11.8	**0.040**
BES	32.2 ± 10.7	31.2 ± 9.9	0.64	37.1 ± 9.7	31.5 ± 10.3	**0.003**
TEMPS-A						
Depressive	9.6 ± 3.4	10.5 ± 3.7	0.18 *	12.1 ± 3.6	10.4 ± 3.4	0.009 *
Cyclothymic	8.3 ± 3.9	7.0 ± 5.0	0.13 *	10.0 ± 4.5	6.9 ± 4.2	<0.001 *
Hyperthymic	10.3 ± 4.8	9.4 ± 5.0	0.42 *	8.2 ± 3.9	9.3 ± 4.8	0.52 *
Irritable	5.1 ± 4.8	5.1 ± 4.1	0.85 *	7.2 ± 3.9	5.4 ± 3.9	0.008 *
Anxious	9.5 ± 4.7	11.8 ± 6.2	0.07 *	13.2 ± 5.4	10.6 ± 5.5	0.009 *

* Mann–Whitney U-test; Legend: BDI: Beck Depression Inventory; STAI: State-Trait Anxiety Inventory; BES: Binge eating scale; TEMPS-A: Temperament Evaluation of Memphis, Pisa, Paris, and San Diego Auto-questionnaire.

**Table 3:** Variables associated with the risk of attrition by the period of attendance to the obesity unit in a multiple logistic regression analysis.

	**Overall Risk of Attrition**		
	**OR; 95% CI**	**Beta**	***p***
BES	1.02; 0.99–1.05	0.022	0.16
TEMPS-A Cyclothymic	1.12; 1.03–1.22	0.117	**0.006**
TEMPS-A Irritable	0.97; 0.89–1.06	−0.028	0.53
	**Risk of attrition during the pandemic period**		
	**OR; 95 CI**	**Beta**	***p***
Males (%)	3.50; 1.04–11.7	1.252	**0.040**
Age of weight gain (years)	0.96; 0.93–1.01	−0.036	0.06
Waist circumference (cm)	1.02; 0.98–1.07	0.023	0.28
	**Risk of attrition outside the pandemic period**		
	**OR; 95 CI**	**Beta**	***p***
BDI	1.05; 0.94–1.17	0.042	0.37
STAI-state	0.97; 0.91–1.02	−0.029	0.23
BES	1.03; 0.98–1.08	0.023	0.17
TEMPS-A Depressive	1.01; 0.87–1.18	0.012	0.87
TEMPS-A Cyclothymic	1.13; 1.00–1.27	0.122	**0.05**
TEMPS-A Irritable	1.01; 0.89–1.15	0.011	0.83
TEMPS-A Anxious	1.03; 0.93–1.13	0.024	0.62

Legend: BDI: Beck Depression Inventory; STAI: State-Trait Anxiety Inventory; BES: Binge eating scale; TEMPS-A: Temperament Evaluation of Memphis, Pisa, Paris, and San Diego Auto-questionnaire.

## Data Availability

Anonymized data are available upon reasonable request to the last author.

## Supplementary Materials

The following supporting information can be downloaded at: https://www.mdpi.com/article/10.3390/jcm11030862/s1, Table S1: Characteristics of the cohort by the period of follow-up. Table S2: Attrition by the period of attendance to the obesity unit.
