# Supplementary material for: In Patients with Obesity, Are Affective Temperaments Associated with Attrition? An Evaluation during and before the SARS-CoV-2 Pandemic
Source: J Clin Med. 2022 Feb 7;11(3):862. doi: 10.3390/jcm11030862 (PMC8836900; doi:10.3390/jcm11030862)
Supplement: Supplementary file 1 [file jcm-11-00862-s001.zip › jcm-1531341-supplementary.pdf]

Supplementary Materials

Supplementary Table S1. Characteristics of the cohort by the period of follow-up.

	Followed-up during the pandemic period n = 89	Followed-up outside the pandemic period n = 122	P
Clinical variables			
Age (years)	48.2 ± 15.5	45.7 ± 15.2	0.24
Males (%)	20.2	23.0	0.64
Residing in a rural area (%)	18.0	17.2	0.89
Active smoker (%)	18.0	15.6	0.64
Living alone (%)	46.1	48.4	0.74
Not working/retired (%)	46.1	41.8	0.54
Secondary schools/graduation (%)	70.8	63.9	0.30
Adverse life events (%)	62.9	77.1	**0.025**
Multiple weight cycling (%)	58.4	42.6	**0.023**
Age of weight gain (years)	26.9 ± 13.5	26.3 ± 14.2	0.75
Weight (kg)	97.5 ± 14.1	99.8 ± 17.3	0.31
Height (cm)	163.6 ± 7.8	163.9 ± 8.2	0.75
BMI (kg/m^2)	36.4 ± 4.6	37.0 ± 4.9	0.37
Waist circumference (cm)	114.0 ± 11.9	115.0 ± 12.2	0.53
Neck circumference (cm)	37.5 ± 4.0	38.4 ± 4.1	0.09
Psychological questionnaires			
BDI	7.8±5.9	10.6 ± 5.9	**<0.001***
STAI-state	45.1±11.6	48.2 ± 11.9	0.06
STAI- trait	46.2±10.4	50.3 ± 11.2	**0.008**
BES	31.6±10.2	34.3 ± 10.4	0.06
TEMPS-A			
Depressive	10.1±3.6	11.2 ± 3.6	0.05*
Cyclothymic	7.5±4.6	8.4 ± 4.6	0.16*
Hyperthymic	9.7±4.9	8.7 ± 4.4	0.28
Irritable	5.1±4.4	6.2 ± 4.0	**0.014***
Anxious	10.9±5.7	11.9 ± 5.6	0.20*

*Mann-Whitney U-test; Legend: BDI: Beck Depression Inventory; STAI: State-Trait Anxiety Inventory; BES: Binge eating scale; TEMPS-A: Temperament Evaluation of Memphis, Pisa, Paris and San Diego Auto-questionnaire.

Supplementary Table S2. Attrition by the period of attendance to the obesity unit

	During pandemic period	Outside pandemic period	p
Number	35	60	
Age (years)	45.9 ± 14.1	43.7 ± 16.1	0.50
Males (%)	34.3	18.3	0.08
Residing in a rural area (%)	20.0	21.7	0.85
Active smoker (%)	14.3	20.0	0.48
Living alone (%)	37.1	53.3	0.13
Not working/retired (%)	40.0	43.3	0.75
Secondary schools/graduation (%)	65.7	63.3	0.82
Adverse life events (%)	51.4	76.7	**0.011**
Multiple weight cycling (%)	51.4	50.0	0.89
Age of weight gain (years)	23.4 ± 11.3	25.2 ± 14.8	0.53
Weight (kg)	98.8 ± 15.2	98.1 ± 17.4	0.85
Height (cm)	164.8 ± 8.2	164.0 ± 8.1	0.65
BMI (kg/m^2)	36.4 ± 5.0	36.4 ± 5.3	0.98
Waist circumference (cm)	117.2 ± 12.5	113.9 ± 11.1	0.19
Neck circumference (cm)	38.1 ± 4.2	37.8 ± 4.2	0.77
Psychological questionnaires			
BDI	6.7 ± 5.7	12.2 ± 5.6	**<0.001***
STAI-state	42.6 ± 10.9	48.8 ± 11.8	**0.014**
STAI-trait	43.6 ± 8.9	52.4 ± 10.2	**<0.001**
BES	32.2 ± 10.7	37.1 ± 9.7	**0.025**
TEMPS-A			
Depressive	9.6 ± 3.4	12.1 ± 3.6	**0.001***
Cyclothymic	8.3 ± 3.9	10.0 ± 4.5	0.08*
Hyperthymic	10.3 ± 4.8	8.2 ± 3.9	0.13*
Irritable	5.1 ± 4.8	7.2 ± 3.9	**0.005***
Anxious	9.5 ± 4.7	13.2 ± 5.4	**0.001***

*Mann-Whitney U-test; Legend: BDI: Beck Depression Inventory; STAI: State-Trait Anxiety Inventory; BES: Binge eating scale; TEMPS-A: Temperament Evaluation of Memphis, Pisa, Paris and San Diego Auto-questionnaire.
